# Methodology to Analyse Three-Dimensional Asymmetries in the Forces Applied to the Pedals in Cycling

**DOI:** 10.1007/s10439-022-03076-y

**Published:** 2022-09-22

**Authors:** Ezequiel Martín-Sosa, Victor Chaves, Elena Soler-Vizán, Juana Mayo, Joaquín Ojeda

**Affiliations:** 1 Departamento de Ingeniería Mecánica y Fabricación, Escuela Superior Técnica de Ingeniería, Universidad de Sevilla, Camino de los Descubrimientos s/n, 41092 Seville, Spain; 2 Departamento de Medicina Física y Rehabilitación, Hospital Universitario Virgen de Valme. Universidad de Sevilla, Ctra. de Cádiz, 41014 Seville, Spain

**Keywords:** 3D pedal forces, Cycling, Asymmetry analysis

## Abstract

The asymmetries study between both legs of the forces applied to the pedals in cycling is important because they may affect the performance of the cyclist or prevent the occurrence of injuries. Studies focused on analysing asymmetries in forces tend to consider only the effective force, disregarding the three-dimensional nature of the force. Furthermore, these studies do not analyse the possible physical or neurological causes that may have led to the appearance of the asymmetries. This paper presents a methodology to carry out three-dimensional analysis of the asymmetries of the forces applied in both pedals and discriminate the possible sources of these asymmetries. Seven participants, amateurs and without pathologies, were analysed. Two commercial pedals were instrumented to measure the three components of the force applied to each pedal. The Normalized Symmetry Index (NSI) and the Cross Correlation Coefficient (CCC) were used for the asymmetries analysis. Results showed that both indexes need to be used in conjunction to analyse the causes of asymmetry in the pedal forces from a 3D perspective along the pedal cycle. The NSI is an index that makes it possible to evaluate asymmetry by considering only the value of the force applied by each leg at each instant. The CCC makes it possible to evaluate whether the temporal evolutions of the forces applied by each leg are similar. Preliminary results suggest that the proposed methodology is effective for analysing asymmetries in the forces in a pedalling cycle from a three-dimensional point of view. Forces in the sagittal plane showed a high level of symmetry. The lateral-medial force presented the highest level of asymmetry due to the difference in the magnitudes of the applied forces by both legs and the existing time shift between the two force patterns. The results of this work will allow for more complete and accurate three-dimensional dynamic analyses of the lower body during pedalling.

## Introduction

An important concept in cycling is pedalling performance,^5^ associated with the ability to apply an effective, power-generating pedalling force.^4^ This performance can be highly influenced by the degree of asymmetry in the application of forces on the two pedals.^8^ In addition to purely mechanical considerations, asymmetries in pedalling can have a biomechanical effect, related to gestures and postures that can lead to overloading of muscles and joints resulting in possible injuries to the cyclist.^2,34^

Focusing on the mechanical aspect, the asymmetries study can be carried out by means of a kinematic or kinetic analysis. To the best of our knowledge, the asymmetries analysis in kinematics is not very extended, highlighting the studies carried out by Pouliquen *et al.* ^30^ and Edeline *et al.*^18^ in which the existence of asymmetries for the 3D kinematics of the lower body is analysed. The asymmetries study in cycling kinetics is more extended due to the direct relationship between effective force, power and performance.^2,3,6,7,9–11,14,17,24,31,33,34^ In this field, the asymmetries analysis is usually based only on the power, torque or effective force applied to each pedal.^2,3,6,7,9–11,14,17,24,31,33,34^ The most relevant of these studies are described as follows. Bini *et al.*^7^ analysed asymmetries in the torque applied to the crank every 5 km in a 20 km test. Smak *et al.*^34^ conducted a study analysing the effect of cadence on the presence of asymmetries in the torque exerted on the crank. Kell *et al.*^24^ analysed the effect of using a cycle ergometer to reduce existing asymmetries in bicycle power output. These studies do not consider the 3D character of the forces, ignoring the effect that the other force components may have on the asymmetries occurrence. To the best of our knowledge, there are no studies in the literature that study asymmetry considering force outside the sagittal plane.

The use of ratios^12,13,25,29^ for the gait asymmetries analysis is common in the literature, with the ratio proposed by Patterson *et al.*^29^ being one of the most widely used by the scientific community. This ratio is an adaptation of the ratio developed by Cuk *et al.*^13^ which was implemented to estimate bone length asymmetries. The ratio proposed by Patterson *et al.*^29^ provides correct results when asymmetry is studied at specific points in the pedalling cycle, such as the instant of greatest or least application of effective force on the pedal or the instant when the hip flexion reaches a peak. However, it has limitations if the temporal evolution of the asymmetries for all the instants that make up the pedalling cycle is evaluated, especially when the variables analysed have values close to zero, as demonstrated by Pouliquen *et al.*^30^ To avoid these limitations, Gouwanda *et al.*^22^ and Pouliquen *et al.*^30^ made modifications to this ratio, calling it the Normalized Symmetry Index (NSI). The modifications focused on normalising the value of the variable so that it would never have a value close to zero. These modifications make it possible to correctly evaluate any instant of the pedalling cycle. In addition, other less common ratios in the asymmetries study, such as the cross-correlation coefficient^26^ (CCC), are also proposed in these studies. This coefficient is used to check whether there is similarity in the time pattern of the variables of one leg with respect to the other. To the best of our knowledge, these two ratios, NSI and CCC, have not been used to the asymmetries study in the three force components applied to the pedal, they have only been used in the study of asymmetries in the cycling^30^ and human gait kinematics.^22^

This paper establishes the following hypotheses as starting points. First, the asymmetries analysis in pedalling forces only in the sagittal plane neglects valuable information for the 3D biomechanical pedalling analysis. This hypothesis is based on the fact that those force components that are not analysed may be presenting asymmetries, which can have a significant impact on the performance and health of the participant.^8^ Secondly, the asymmetries analysis at every moment of the pedalling cycle, and not just at one single instant, allows to know more details about the differences between both legs. Third, asymmetry in the pedalling forces is not only due to differences in the values of the forces applied by each leg. In order to analyse the validity of these hypotheses, the main objective was established as an asymmetry analysis of the three components of the forces applied to each pedal during pedalling. For this purpose, the three-dimensional forces applied to the pedals by both legs during pedalling were experimentally recorded using equipment developed by the authors.^28^ This equipment is attachable to any conventional bicycle and pedals and, due to its small size and location, it does not alter the natural pedalling of the cyclist. The NSI and CCC coefficients were used to study the asymmetry temporal evolution in the forces exerted during pedalling.

## Materials and Methodology

### Participants

This study included 7 male participants, adults, and amateurs, with a mean age of 27.96 ± 3.98 years, a mean height of 1.77 ± 0.12 m and a mean mass of 79.01 ± 5.22 kg. The sample size was based on previous studies found in the literature.^9^ The main objective of the work was to develop a methodology to analyse the asymmetries in the pedalling forces in a general and complete way throughout the cycle.

The inclusion/exclusion criteria were as follows:

- Participants over 18 years of age.
- No diagnosed locomotor or cardiopulmonary pathology that would prevent the test from being performed.
- No lower limb dysmetry greater than 5 mm.
- Participants size fits the test bike size.
- Participants intrinsic Q-factor no greater than the bicycle standard Q-factor of 285 mm.
- Regular use of bicycles as a means of transport, but not for sporting or improving the performance.

To assess the discrepancy between lower limbs, the direct method with a tape measure was used, with measurement in the supine position from the anterior superior iliac spine to the lateral malleolus, comparing both legs. Dysmetry was considered to have no clinical impact when it was less than 5 mm. Radiological measurements were not used for ethical and subject safety reasons. All subjects had dysmetry of less than 5 mm and were therefore included in the study.

Participants signed a consent form approved by the Ethics Committee Andalusian Biomedical Research Ethics Platform (approval number 20151012181252). Each participant was asked about their dominant leg, such as the leg used to kick a ball.^8,20,21,27,34^

### Instrumentation

**Figure 1:**
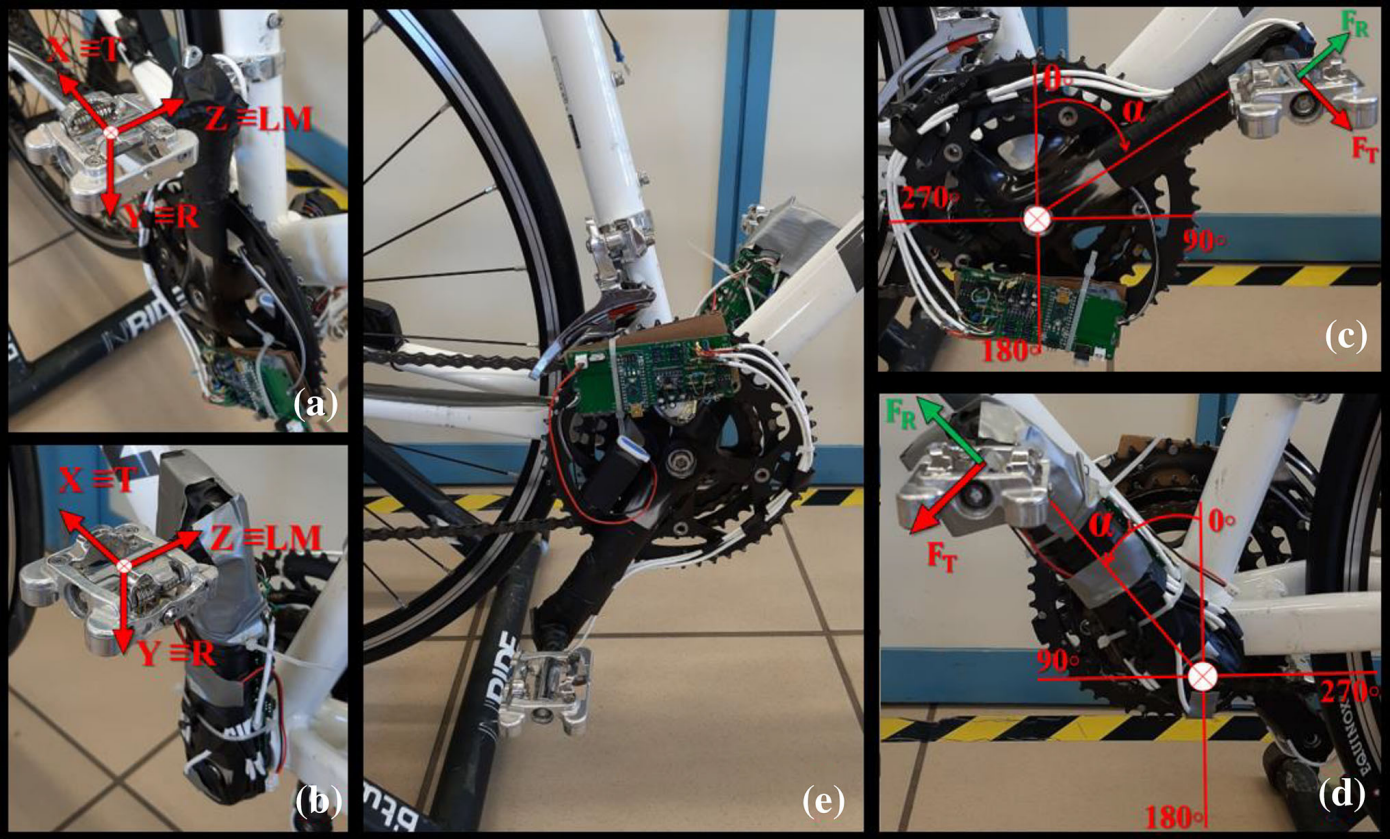
(a) Right Pedal Coordinate System. T: Tangential direction. R: Radial direction. LM: Lateral-medial direction. (b) Left Pedal Coordinate System. (c) Right crank rotation angle, α, respect to a vertical line and forces components F_T_ y F_R_ applied to the left pedal. (d) Left crank rotation angle, α, respect to a vertical line, and forces components F_T_ y F_R_ applied to the right pedal. (e) Measurement Equipment.

The applied force in the pedal was assumed to be pointwise at the centre of the pedal. A system of axes attached to the pedal axle and the crank was defined to estimate the three components of this force, following the methodology used by Martín-Sosa *et al.*^28^ (Fig. 1a and b). The X-axis was defined in the direction tangential to the path of the crank attachment point to the pedal axle, the Y-axis in the direction of the crank axle and the Z-axis in the direction of the pedal axle (Fig. 1a). This definition of the coordinate system was used for both the left and right pedal, due to the plane of symmetry defined by the bicycle frame (Fig. 1b). The following nomenclature was used to define the components of the forces: the force applied in the X direction was called tangential force, F_T_, because it is tangent to the trajectory of the end of the crank. The force applied in the Y direction was called radial force, F_R_, which follows the direction of the crank axis. These two force components are in the sagittal plane, parallel to the bicycle frame (Fig. 1c and d). Finally, the component of the force in the Z-direction, which follows the direction of the pedal axle, was termed the lateral-medial force, F_LM_. According to these definitions, only the F_T_ force produces an effective moment which rotates the crank. The other two forces, F_R_ and F_LM_, do not produce any effective moment on the crank. The position of the crank was defined by the angle α, formed from this and the vertical, where 0° is the angle corresponding to the top dead centre, TDC (Fig. 1c and d). With the crank at TDC, the positive direction of the forces is as follows: F_T_ positive in the forward direction. F_R_ positive in the upper direction. F_LM_ positive in the medial direction.

Measurements of the three components of the force applied to each pedal were carried out using an electronic device developed by Martín-Sosa *et al.*^28^ (Fig. 1e), which can be attached to a conventional bicycle and pedals. This equipment used six strain gauges for each pedal, four placed on the pedal axle and two on the crank. The gauges were arranged in pairs, in a half Wheatstone bridge configuration. The maximum error recorded for each force component was 3.27% for the tangential component, 4.21% for the radial component and 1.34% for the lateral-medial component.^28^ The two measuring devices were mounted on a commercial bicycle, model Triban 500, which was anchored to an Elite Roller training roller. The equipment placed in the described locations did not alter the natural pedalling of the participants. WPD-M17C clipless pedals were used.

### Test Conditions

Participant trials were conducted with a pedalling power of approximately 150 W and at a pedalling cadence of approximately 60 rpm. The pedalling conditions were defined according to the characteristics of the sample used. Each participant was asked about the time spent on the bicycle during their usual riding, the frequency of use of the bike and the approximate average speed.

In addition to this information, A pulse oximeter and individual maximum frequency measurement were also used, together with a basic health questionnaire consisting of the following items:

- Personal history
- Cardiopulmonary auscultation. Oxygen saturation and resting heart rate.
- Measurement of lower limb length
- MRC scale for assessment of dyspnoea with exercise. This scale was chosen because it is a sample of non-athletes.

The chosen participants were not used to pedalling at high cadences and high power, so the test conditions were adapted to their physical characteristics, to avoid the effects of fatigue. For this reason, 150 W and 60 rpm were chosen. An increase in cadence to 90 rpm and power to 200 W produced the appearance of fatigue in the participant in a few minutes, affecting pedalling. If only the cadence was increased, leaving the pedalling power at 150 W, the subject would notice little resistance in pedalling, causing difficulty in maintaining the desired cadence and, consequently, generating pedalling cycles that were not homogeneous due to the constant increase and reduced pedalling cadence. If the pedalling power was increased and the cadence was maintained, the subjects began to report muscle fatigue, especially in the quadriceps, due to the application of force. This fact would lead the subject to adopt a different pedalling posture to relieve muscle pain, thus causing a change in both the pedalling movement and the application of forces. For all these reasons, 150 W and 60 rpm were finally chosen. Anyhow, during the recording of the forces, participants were asked to assess their level of fatigue. In all cases, it was reported that there was no physical fatigue.

The decision was made to stablish unique cycling conditions for all participants but affordable for all so as not to introduce another possible biasing variable in the analysis of the results.

The influence of cadence was a very important aspect to consider. The selected power output showed that increasing or decreasing the cadence could significantly alter the pedalling pattern. A very high cadence could result in the participant not being able to maintain a uniform pedalling pattern. Conversely, by decreasing the cadence, participants could become fatigued, as the torque to be achieved was higher.

Shoes were provided to the participants. Several sizes were available so that participants could choose the shoe size that best fit them and thus pedal comfortably. The correct placement of the cleats was verified following a protocol. First, the cleat and shoe recommendations of the manufacturer were followed for the location and orientation of the cleats in the shoes. Then, for each participant, it was verified that the cleats were located on the fat pad at the level of the 1st metatarsal head.^1^ The correct position of both cleats was certified by checking that the tip of the shoe for both feet were the same distance from a reference point. Additionally, cardboard shoe insoles to verify that the cleat orientation in both shoes was identical and correct were used. In short, the correct position of the cleats for each test was verified with considerable precision.

**Figure 2:**
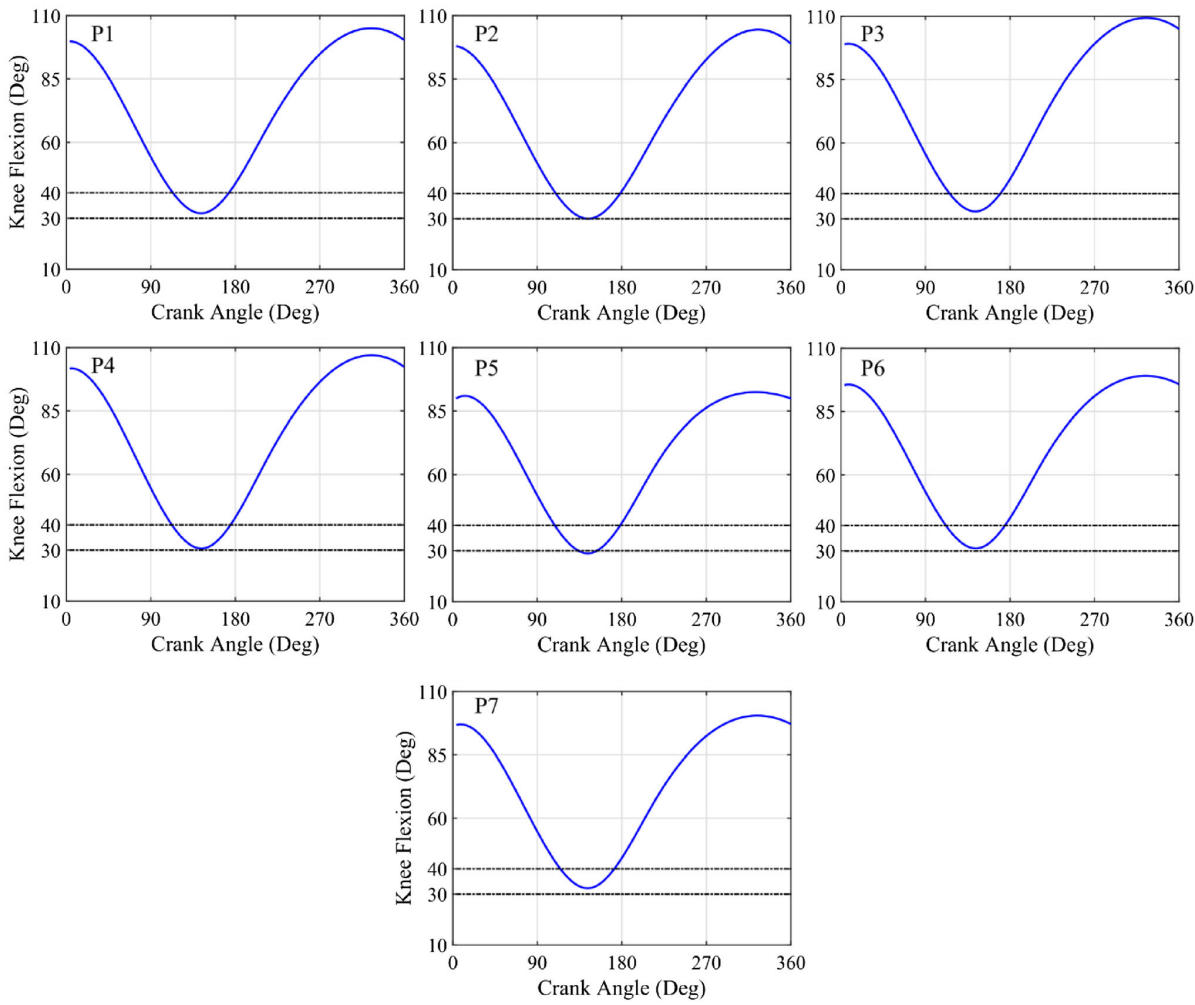
Temporal evolution of the knee flexion angle for the 7 participants after adjusting the saddle height. The dashed horizontal lines delimit the interval defined by Ferrer-Roca.^19^

Saddle height was determined from the flexion angle of the right knee as it was stablished by Holmes^23^ but in a dynamic way. To determine this angle, reflective markers were placed on the greater trochanter, lateral epicondyle and lateral malleolus, all on the right side, and the inverse kinematic problem was solved. To achieve greater comfort for each participant, the saddle height could be slightly modified, provided that the minimum angle of knee flexion was within the range (30°–40°).^19^ A linear relationship between the saddle height and the subject height was not found, since other parameters such as the length of the legs or the flexibility of the muscles have an influence on the flexion angle.^19^ The Fig. 2 showed the knee flexion angle for the seven participants. It can be verified that the minimum flexion angle is within the range defined previously, except for participant 5 who showed a minimum knee flexion of 29°. As this value was out of range by only 1°, the saddle height was left as valid because the subject had reported discomfort with smaller saddle heights.

The duration of each trial was about 15 min, with the first five minutes being used for a light warm-up and adaptation to the power and speed conditions set. Once the saddle height was adjusted and taking into account that one of the inclusion/exclusion criteria for the sample was the compatibility of the size of the partcipant with the size of the bicycle, the participant was allowed to lean on the handlebars in the way that was most comfortable for them. The position of the handlebars could not be altered, which was a limitation of the study. However, in all cases, the participants reported feeling comfortable with the bicycle setup and pedalling conditions. The forces applied to the pedals during the remaining 10 min of the test were measured. The start of each pedal cycle was taken at TDC of the right pedal.

### Analysis of Asymmetries

Analysis of the existence of asymmetries between the dominant and non-dominant leg for each component of the force applied to the pedal was carried out by applying two coefficients to the data collected: firstly, the NSI presented in the work of Pouliquen *et al.*^30^ and Gouwanda *et al.*^22^ which is a modification of the Symmetry Ratio implemented by Patterson *et al.*^29^ Secondly, the CCC developed by Li and Caldwell.^26^

The NSI was used to determine the degree of asymmetry at each instant of the pedal cycle between the dominant and non-dominant side:^22,30^

1 $$NSI\left( t \right) = \frac{{FN_{D}^{C} \left( t \right) - FN_{ND}^{C} \left( t \right)}}{{0.5*\left( {FN_{D}^{C} \left( t \right) + FN_{ND}^{C} \left( t \right)} \right)}}*100\%$$

2 $$FN_{i}^{C} \left( t \right) = \frac{{F_{i}^{C} \left( t \right) - F_{\min }^{C} }}{{F_{\max }^{C} - F_{\min }^{C} }} + 1$$

In Eq. (1), *NSI(t)* represents the normalised rate of symmetry between dominant and non-dominant leg for each time instant *t*. ${FN}_{D}^{C}(t)$ and ${FN}_{ND}^{C}(t)$ are the normalised force values for the C component (tangential, radial or lateral-medial) corresponding to instant *t* for the dominant and non-dominant leg respectively. The normalised forces are defined in Eq. (2), where ${F}_{i}^{C}(t)$ is the value of the C component of the force for time instant *t* and leg *i*, where *i* is either leg dominant (D) or non-dominant (ND). ${F}_{max}^{C}$ and ${F}_{min}^{C}$ are the maximum and minimum values respectively for the analysed cycle of the C component of the force, they can belong to either the dominant or non-dominant leg. Normalisation prevents possible singularities from appearing when force values tend to zero. When evaluating the NSI, values close to 0 will represent high symmetries, positive values will reflect a higher force applied with the dominant side while negative values will indicate a higher force applied with the non-dominant side as long as the forces have a positive sign. In the case where the forces are negative, it may be possible to obtain an index with a negative value and yet the value of the component of the dominant force is greater in absolute value than the component of the non-dominant leg. In this work, a threshold value of NSI = 0.1 has been adopted, above which a state of asymmetry between dominant and non-dominant leg components is considered.^9^ From this threshold value, a new Asymmetry During Cycle (ADC) index has been defined, as follows:

3 $$ADC = \frac{{t_{{\left| {NSI} \right| > 0.1}} }}{T}*100$$

where T is the duration of a pedalling cycle and ${t}_{\left|NSI\right|>0.1}$ the time where the index NSI is higher than 0.1 in modulus. This index can be used to determine the percentage of the pedal cycle in which a component of the force behaves asymmetrically.

The CCC^26^ was used to detect similarities in the temporal evolution of the forces applied by the dominant and non-dominant legs. It is defined as follows:

4 $$CCC\left( k \right) = \frac{{c_{{F_{D} F_{ND} }} \left( k \right)}}{{\sqrt {\mathop \sum \nolimits_{t = 1}^{N} \left( {F_{D}^{C} \left( t \right) - \overline{{F_{D}^{C} }} } \right)^{2} *\mathop \sum \nolimits_{t = 1}^{N} \left( {F_{ND}^{C} \left( t \right) - \overline{{F_{ND}^{C} }} } \right)^{2} } }}$$

where:

5 $$c_{{F_{D} F_{ND} }} \left( k \right) = \left\{ \begin{gathered} \mathop \sum \limits_{t = 1}^{N - k} \left( {F_{D}^{C} \left( t \right) - \overline{{F_{D}^{C} }} } \right)*\left( {F_{ND}^{C} \left( {t + k} \right) - \overline{{F_{ND}^{C} }} } \right) + \hfill \\ + \mathop \sum \limits_{t = N - k + 1}^{N} \left( {F_{D}^{C} \left( t \right) - \overline{{F_{D}^{C} }} } \right)*\left( {F_{ND}^{C} \left( {t - N + k} \right) - \overline{{F_{ND}^{C} }} } \right), if\;k \in \left[ {1,N} \right] \hfill \\ \mathop \sum \limits_{t = 1}^{N - k} \left( {F_{D}^{C} \left( t \right) - \overline{{F_{D}^{C} }} } \right)*\left( {F_{ND}^{C} \left( t \right) - \overline{{F_{ND}^{C} }} } \right),\quad if\;k = 0 \hfill \\ \end{gathered} \right.$$

In Eq. (4), *CCC(k)* represents the cross-correlation coefficient for a time shift *k* between the dominant and non-dominant side signal. For this purpose, the measurements of the signals applied on the non-dominant side were put in phase with the signals applied on the dominant side to synchronise both signals at the start of the pedal cycle, TDC. For the set of equations, ${F}_{D}^{C}(t)$ and ${F}_{ND}^{C}(t)$ represent the C component (tangential, radial or lateral-medial) of the forces applied by the dominant and non-dominant leg at each instant of time *t*. *N* is the number of instants that compose the pedalling cycle. $\overline{{F }_{D}^{C}}$ and $\overline{{F }_{ND}^{C}}$ represent the average value of the C-component of the force exerted by the dominant and non-dominant leg over the analysed pedalling cycle, respectively. ${\tau }_{lag}$ is defined as the pedal angle delay and indicates the lag of the non-dominant component with respect to the dominant component. It corresponds to the value of *k* at which the correlation coefficient *CCC(k)* is maximum. A ${\tau }_{lag}$ positive value indicates that the non-dominant leg component is shifted forward in the pedal cycle relative to the dominant leg. The *CCC(k)* oscillates in the interval [− 1, 1], with the maximum value corresponding to the best overlap between the forces on the dominant side and the non-dominant side. The CCC (k = 0) will give an indication of pattern similarity between the two sets of data. If there was a time (or phase) shift between two-time series that have similar patterns, the magnitude of this shift could be found by assessing CCC(k) at different values of k. An objective measure of the time shift is the k at which CCC(k) is maximized. The 95% confident interval (CI) of the highest CCC can be calculated by the following expression:

6 $$CI_{95\% } = \left[ {\frac{{e^{{2h_{1} }} - 1}}{{e^{{2h_{1} }} + 1}} , \frac{{e^{{2h_{2} }} - 1}}{{e^{{2h_{2} }} + 1}}} \right]$$

where

7 $$\begin{array}{*{20}c} {h_{1} = \frac{1}{2}\ln \left( {\frac{1 + CCC}{{1 - CCC}}} \right) - \frac{1.96}{{\sqrt {N - 3} }}} \\ {h_{1} = \frac{1}{2}\ln \left( {\frac{1 + CCC}{{1 - CCC}}} \right) + \frac{1.96}{{\sqrt {N - 3} }}} \\ \end{array}$$

The ${CI}_{95\%}$ of CCC(k) can be converted to corresponding values in the time domain to determine if the phase shift between the two set of data is statistically significant. It will be statistically significant if the value of CCC(k = 0) does not belong to the ${CI}_{95\%}$ ^26^.

Due to the fact that no values have been found in the literature, thresholds have been established to consider asymmetries in the strength patterns. Thus, maximum CCC values above 90% and an offset below 10° would indicate that there is a great symmetry between the two strength patterns (dominant and non-dominant leg). In any case, the proposed methodology is general and independent of the threshold values defined to analyse the asymmetries.

## Results

**Figure 3:**
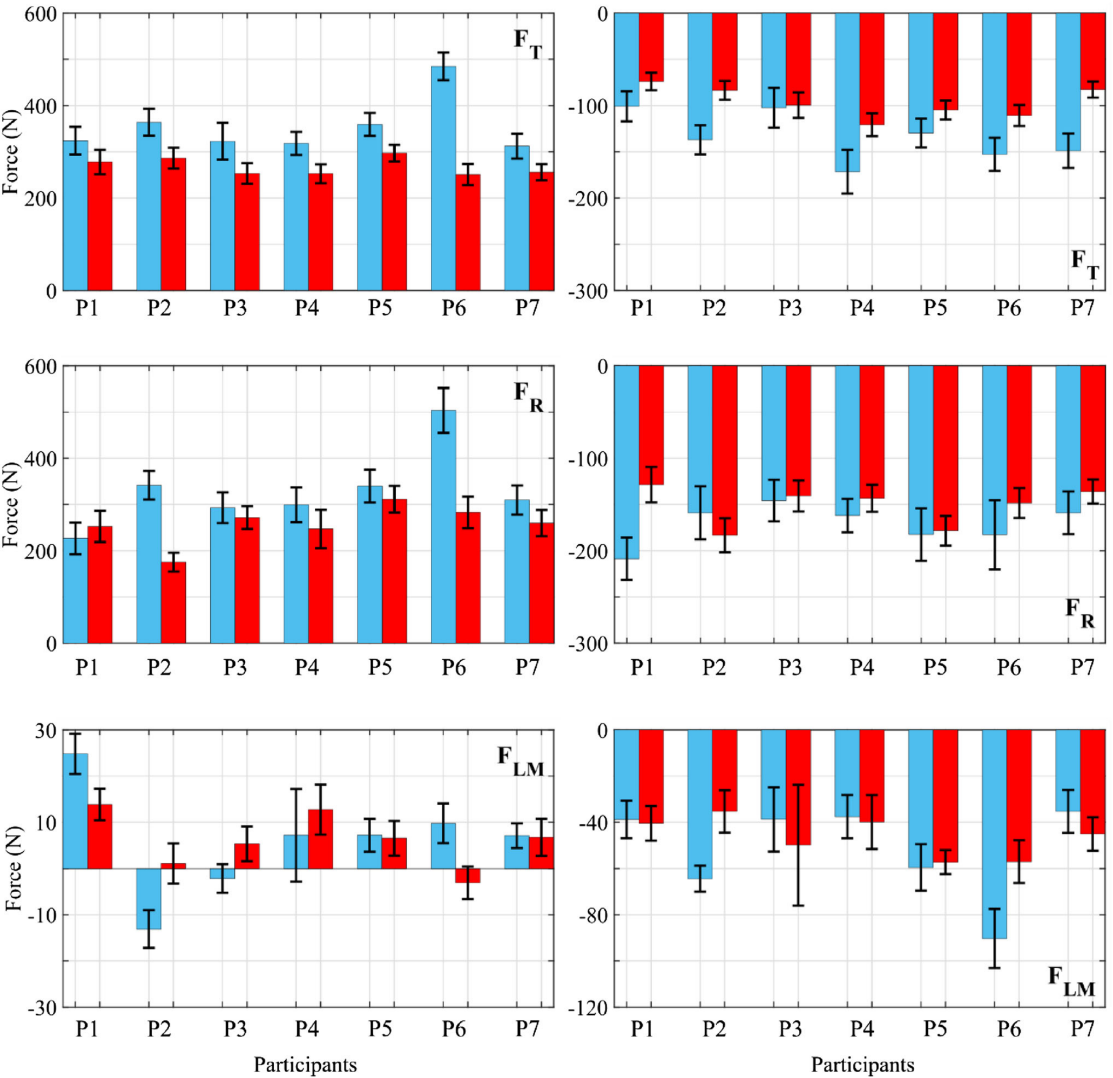
Differences between dominant and non-dominant leg side for the force components applied to the pedals. Left: forces maximum values. Right: forces minimum value. Blue bars: Dominant leg. Red bars: non-dominant leg. F_T_: Tangential force. F_R_: radial force. F_LM_: Lateral-Medial force. Data were presented as mean & ± SD.

Figure 3 showed the maximum and minimum values for the three force components applied to the pedal for the dominant and non-dominant leg for all participants.

**Figure 4:**
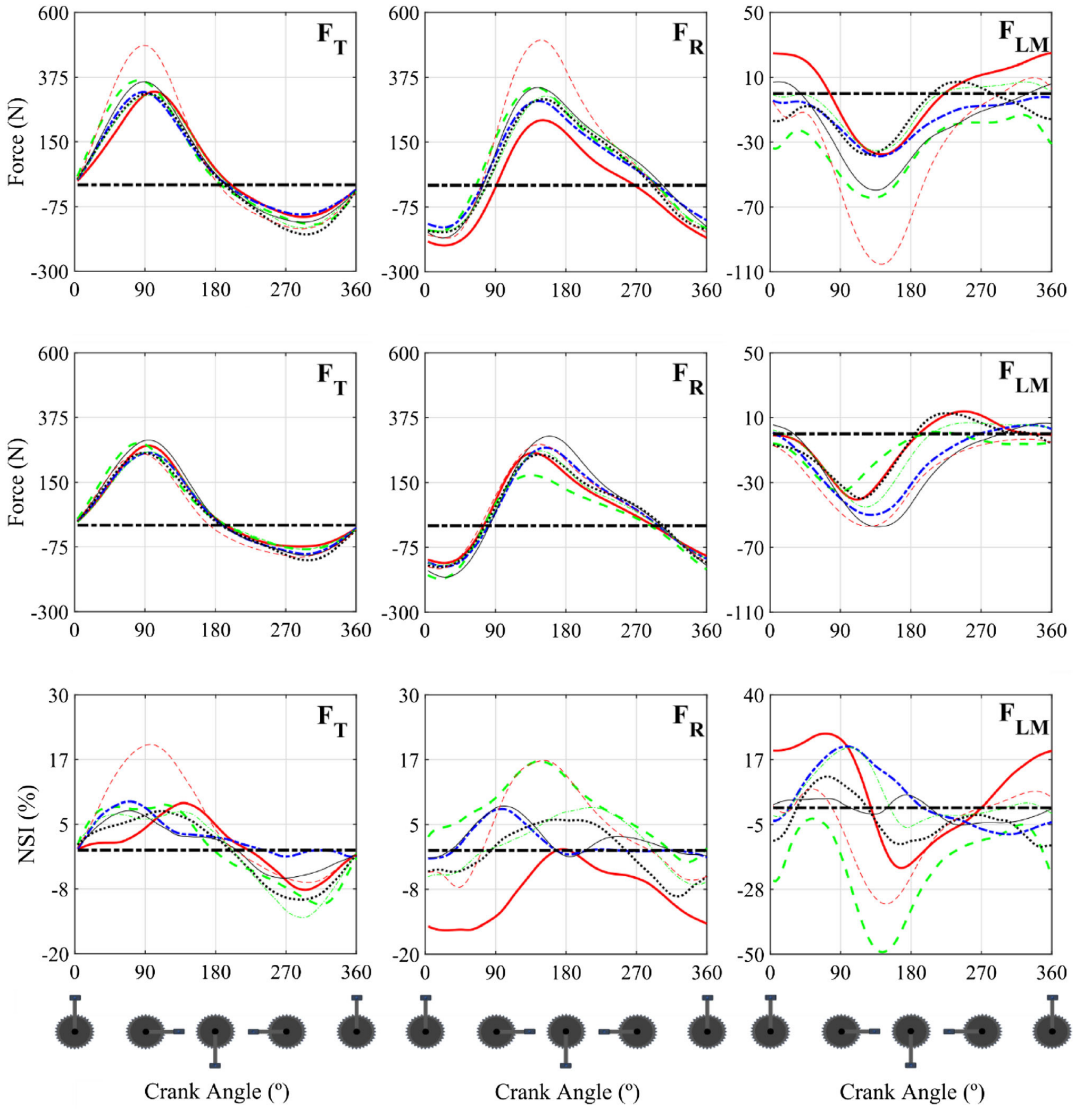
Average forces evolution in a pedalling cycle and temporal evolution of the average NSI index for the three force components. Upper: dominant leg force. Middle: non-dominant leg force. Lower: NSI index. Thick solid red: P1. Thick striped green: P2. Blue thick dotted dashed: P3. Thick black dotted: P4. Solid fine black: P5. Fine striped red: P6. Green fine dot-dashed green: P7. Thick black dot-dashed black: 0 N line. F_T_: Tangential force. F_R_: radial force. F_LM_: Lateral-Medial force.

Figure 4 showed the angle evolution of the average forces applied on the pedal for the dominant and non-dominant leg and the temporal evolution of the average NSI index for the three force components for the seven participants.

Table 1 showed the average values and deviations of the ADC index for the three components of the forces applied to the pedal for each of the participants.

**TABLE 1:** ADC values for the force components of the 7 participants.

	Force comp	Participant						
		P1	P2	P3	P4	P5	P6	P7
ADC	F_T_	14.36 ± 12.34	17.80 ± 12.37	13.53 ± 11.54	17.79 ± 12.83	6.56 ± 9.00	34.97 ± 7.67	23.23 ± 11.14
	F_R_	39.91 ± 12.74	33.70 ± 9.02	11.22 ± 11.77	16.72 ± 13.30	8.69 ± 10.45	31.12 ± 8.90	9.21 ± 11.28
	F_LM_	68.23 ± 11.45	69.45 ± 15.59	49.17 ± 15.86	46.65 ± 19.52	25.39 ± 16.21	34.50 ± 6.96	32.02 ± 11.53

Data were presented as mean & ± SD

*F*_*T*_ Tangential force, *F*_*R*_ radial force, *F*_*LM*_ lateral-medial force, *Pi* Participant *i*

## Discussion

Firstly, the temporal evolution of the forces recorded on the pedals was studied for the seven participants. Although the amateur nature of the participants could *a priori* be a limitation of the study, a sample was defined to represent subjects who use the bicycle on a daily basis as a means of transport because it is a population group that, in the opinion of the authors, is becoming increasingly important in the world today. Therefore, the authors thought that this population may be of interest when analysing asymmetries and, consequently, possible injury risks. In any case, the force curves performed during pedalling were analysed and it was found that these were very much in agreement with previous studies.^15,16^ Therefore, even though the participants were amateur, their pedalling technique was good enough to consider that this could be a distorting factor. Then, each force component was then analysed separately.

F_T_ analysis showed a temporal pattern qualitatively similar for both legs (Fig. 4). However, the maximum modulus forces occurred in the dominant leg (Fig. 3). The instants of maximum F_T_ also produced the maximum dispersion in the results. In any case, the maximum dispersion obtained in the results was around the 15% at the moments of maximum F_T_. The error of the measuring equipment was less than 5 N, which represent around the 10% of the variability. Therefore, most of the variability observed was due to the execution by the participants. From the point of view of inter-subject analysis, some variability in the results was also observed due to oscillations around the set pedalling conditions. Thus, although the conditions of 150 W and 60 rpm were fulfilled on average, oscillations around these parameters were observed instantaneously, especially when instants of maximum force were reached.

F_R_ study results showed similar conclusions to F_T_ in terms of qualitative analysis, relationship between the dominant and non-dominant leg although the maximum dispersion reached slightly higher values, around 20–25%.

The lateral-medial component temporal evolution showed greater variability in the inter-subject analysis. This happens because the force values recorded in this direction were an order of magnitude smaller than the forces in the sagittal plane. Therefore, the dispersion commented in the results affected more this component. Nevertheless, a similar qualitative evolution was observed in all participants.

**Figure 5:**
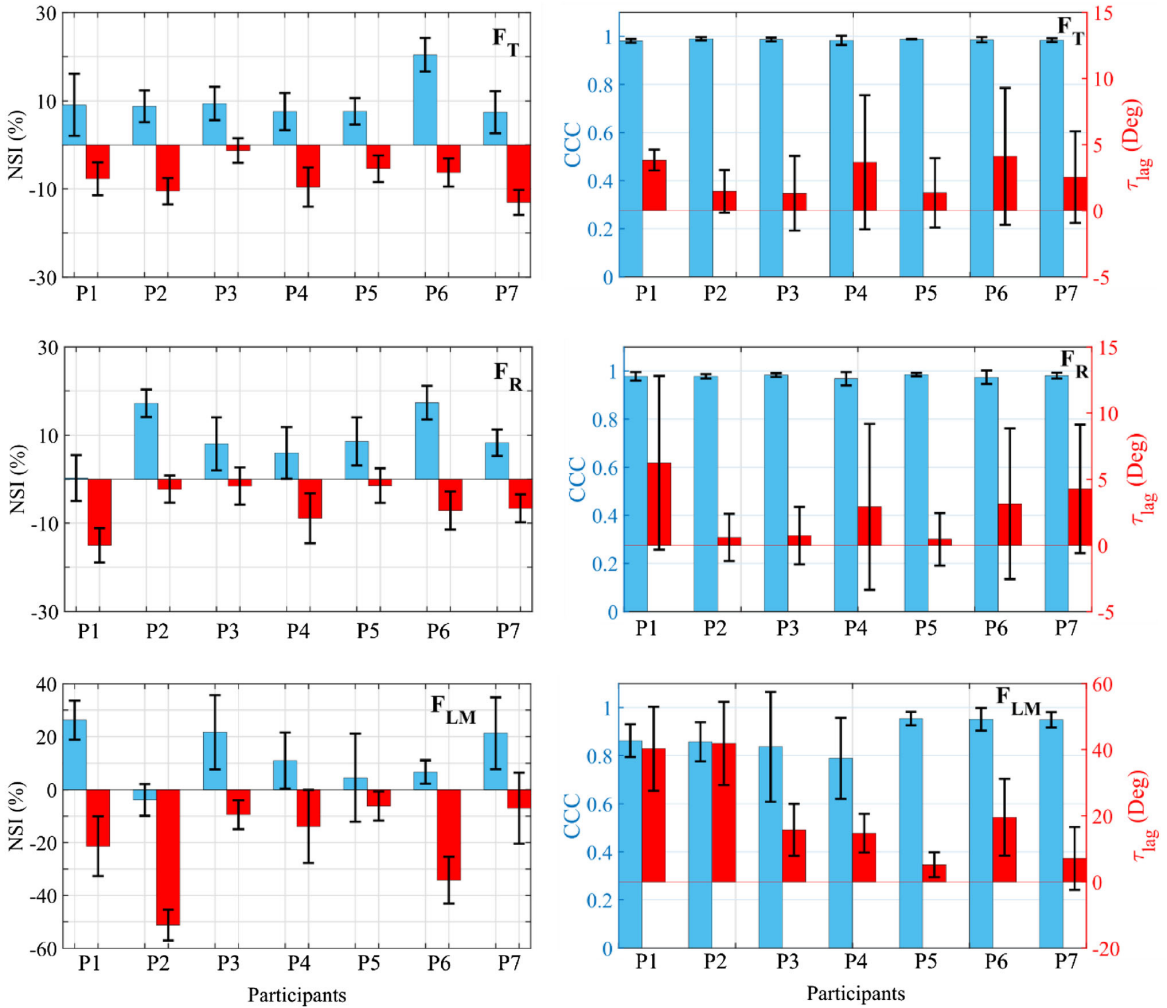
NSI and CCC values for the force components recorded at the pedals. Left: NSI values. Right: CCC and ${\tau }_{lag}$. NSI graphics: Blue bars: NSI maximum values. Red bars: NSI minimum values. CCC and ${\tau }_{lag}$ graphics: Blue bars: CCC values. Red bars: ${\tau }_{lag}$ values. F_T_: Tangential force. F_R_: radial force. F_LM_: Lateral-Medial force. Data were presented as mean & ± SD.

The NSI temporal evolution analysis in Fig. 4 showed that the F_T_ presented a high level of symmetry throughout the cycle, with the NSI not exceeding 16% in modulus except in participant 6. However, the results showed a high level of variability across all participants (see Fig. 5). The NSI modulus reached the peak in two characteristic areas: for a crank angle of about 90° and for a crank angle of about 300°. In the first case, the force was decreasing with the same rate of change in both legs, which was very high. However, in the dominant leg it happened a little bit later, which causes the difference of forces between dominant and non-dominant leg to be very high. The second case occurred when the F_T_ modulus reached a peak while the leg was "opposing" the movement of the pedal. In this case, the non-dominant leg exerted less force in absolute value because the gravitational component was compensated by the inertial force and, in addition, by the force applied to the leg, which was greater in the dominant leg than in the non-dominant leg.

F_T_ was positive in both legs until after the bottom dead centre, with dominant leg component being greater. In the second part of the cycle there came a point where both forces were negative and thereafter the component of the dominant leg was greater in modulus. At this point there was a change of sign in the NSI index. In the first instance, a positive NSI value implied that the force in the dominant leg was greater than in the non-dominant leg and *vice versa*.^22,30^ However, according to the mathematical index definition (Eqs. 1 and 2), there may have been cases where the physical interpretation of the numerical value leads to an erroneous analysis. This happened in cases where the forces on both legs were negative, but the dominant component was greater in modulus, as occurs in F_T_ of participant 1 when the angle α reached a value of 227°. In this case, the NSI had a negative value, but a greater force, in modulus, was being exerted with the dominant leg than with the non-dominant leg. To the knowledge of the authors, this fact was inherent to any procedure in which the variables were normalised to avoid possible singularities in the values of the asymmetry index. Therefore, some additional information was required to make a complete and unequivocal physical interpretation of the NSI and, in general, of any index of asymmetry that involved a normalisation of the variables. The approach proposed in this work was the NSI modulus analysis complemented with data from the force values involved in its calculation at each instant. Thus, for example, F_T_ of participant 1, for a crank angle of 270°, showed an NSI in modulus of 5.8, which implied a high level of symmetry, with the force applied with the dominant leg being greater than with the non-dominant leg.

In relation to F_R_ asymmetry level showed that it was slightly higher, reaching in some cases the maximum values in modulus of the NSI of 20%, except in participant 6, which reached 22% (Fig. 5). The NSI maximum value for F_R_ was reached for an angle α between 90° and 180°. In this interval of the cycle, F_R_ maximum values were reached, which were different in both legs and, moreover, were out of phase. These two facts caused an increase in the asymmetry level as mentioned above. A greater variability in asymmetry was also observed in the inter-subject analysis (Fig. 5) because of morphological asymmetries, the lack of pedalling skills of the participants, etc. For example, in the case of participant 1, the NSI was negative for most of the cycle. The NSI maximum value in modulus was reached for an angle α of approximately 45°. The leg performed a compressive force on the crank at this moment. Although the NSI value was negative, the previously mentioned had to be taken into account and the force values in the dominant and non-dominant legs had to be analysed. Thus, a greater force in modulus exerted by the dominant leg was observed.

F_LM_ is the one which shows the highest levels of asymmetry, reaching NSI values above 30% in many cases, and variability (see Fig. 5). The maximum values of the index occurred at two instants. Firstly, for values of α between 45° and 90°, instants when the forces of both legs had a very high rate of variation but were slightly delayed in time. In this way, the NSI reached one of its maximums during the cycle. Secondly, when the force of the dominant leg reached its maximum in modulus, for a value of α between 135° and 180°. Again, the gap between the two forces caused the NSI to reach a maximum in modulus at that point. In a similar way, the NSI value was negative, but the force exerted by the dominant leg in modulus was greater than that exerted by the non-dominant leg. This higher value of the asymmetry level in the F_LM_ could be due to two main reasons. Firstly, as mentioned above, the lack of experience of the participants, since they were all amateurs without a refined knowledge of pedalling technique. However, to confirm this explanation, the results would have to be compared with those obtained in professional cyclists. Secondly, the control of the forces applied in the lateral direction was more complicated from a biomechanical point of view, this force component being particularly sensitive to factors such as the configuration of the bicycle (saddle height, Q factor, etc.), the morphology and the pedalling technique of the participant. In this way, the saddle height was adjusted for each participant using the methodology developed by Holmes *et al.*^23^ However, the Q factor was kept constant because it was, in general, a standard and fixed parameter defined by the bicycle manufacturer.

In order to analyse whether the proposed methodology allows validation of the second hypothesis of this study, an index was defined based on the NSI that provides information on the percentage of the pedalling cycle in which the forces performed by both legs were asymmetrical. For this purpose, a threshold value of the NSI was defined in absolute value taken from the literature.^9^ The defined index (ADC) is shown in Table 1 for all participants. When analysing the results, it should be borne in mind that they are obtained for each participant from all the cycles carried out, in which there is a dispersion. The F_T_ component shows, in general, a fairly symmetrical behaviour throughout the cycle. The asymmetry occurs in a percentage of the cycle lower than 20% in all cases except for participants 6 and 7. The F_R_ component presents a higher degree of asymmetry since only 4 participants present asymmetry in a phase of the cycle of less than 20%, the other 4 participants presenting asymmetries that represent between 30 and 40% of the pedalling cycle. Finally, the lateral component of the force is the one with the greatest asymmetry. In participants 5–7 the asymmetry is 25–35% of the pedal cycle. In participants 3 and 4 it is between 45 and 50% of the cycle and participants 2 and 3 reach almost 70% of the pedal cycle with asymmetric behaviour.

The results discussed above suggest that, on a preliminary basis, the proposed methodology allows information to be obtained on the first two hypotheses proposed in this paper. First, the importance of carrying out a three-dimensional asymmetry analysis is highlighted, because, as has been demonstrated, the greatest asymmetries are located in the component of the force not contained in the sagittal plane. Secondly, the asymmetries analysis throughout the pedalling cycle provides a global and complete knowledge of pedalling behaviour.

The CCC complemented the NSI by analysing the similarity between patterns of two variables. The results in Fig. 5 reflected that the forces contained in the sagittal plane were highly correlated with maximum CCC values above 96%. Moreover, this similarity between the force patterns occurred with a minimal offset, never greater than 7°. The correlation of the F_LM_ patterns showed CCC values for participants 1 to 4 below the established threshold of 90%. Analysing the phase difference, all the participants except 5 and 7 have a phase difference of more than 10°. Thus, participants 1 to 4 present a clear asymmetry in the lateral force patterns, since the correlation is low, and the phase shift is high. Participant 6 presents a high correlation between patterns but with a 20° offset, with a high variability in the offset, which indicates in some way that the patterns are not symmetrical. Participants 5 and 7 show high symmetry in lateral force patterns in both correlation and phase shift. In all participants the lag values were positive which meant that the non-dominant leg was shifted forward compared to the dominant leg. From a physiological point of view, these results were the opposite of what was expected. The cerebellum is responsible for directing motor activities, controlling movements linked to both gross and fine motor skills. Transcranial magnetic stimulation studies revealed that the dominant hemisphere of the cerebellum requires less stimulation than the non-dominant hemisphere in eliciting muscle responses from the primary motor cortex.^32^ Therefore, the non- dominant leg forces would be expected to lag behind the dominant leg, requiring more stimulation from the non-dominant hemisphere. Consequently, the results obtained were understood to have a justification based on biomechanical aspects. Firstly, preliminary studies carried out by the authors of this paper suggested that increasing pedalling power the non-dominant force lag increased relative to the dominant leg in the sagittal plane. These results were in agreement with the conclusions obtained from the literature.^32^ The reason for the slightly different results in this work may have been due to the low workload and cadence defined in the test conditions. Thus, the inertial force produced by the dominant leg could produce an advance in the pattern of the non-dominant force. The increase in pedalling workload did not suggest a correlation with the offset in the lateral-medial component. A more in-depth study would be necessary to analyse the behaviour of this force component as a function of pedalling workload.

The results obtained show that, in the case of forces contained in the sagittal plane, the level of symmetry in the force patterns is very high. However, the results preliminarily suggest that the third hypothesis could be accepted in the case of lateral force since asymmetries in the force patterns were observed in 5 of the 7 participants analysed. This fact highlights the need to use both indices when carrying out an asymmetries analysis. Thus, a high NSI value with a high cross-correlation and a minimal offset indicated that the asymmetry is due to differences in the applied forces magnitudes, however, there was a high synchrony in the dominant and non-dominant legs force patterns (e.g. F_T_ of participant 6 or F_LM_ of participant 7). A high NSI value and a low cross-correlation indicates a high asymmetry considering both factors, forces magnitude and synchrony level (example: F_LM_ participants 1 and 2). However, no cases had been detected with a low NSI and correlation value. This indicates that, if a participant is able to perform the same force with both legs, he/she also performs a good synchronisation between both legs.

The results obtained at the force level were consistent with those reported by other authors at the kinematic^30^ and kinetic^14,24,33,34^ level. In addition, the level of symmetry between dominant and non-dominant leg in the sagittal plane is greater than the one obtained in the lateral-medial direction. These results are similar to those reported by Pouliquen and colleages^30^ in the kinematic level. However, no references were found regarding symmetry analysis in the kinetic level from a 3D perspective. Therefore, our understanding is that it is necessary to develop this line of research. This asymmetry degree observed in the lateral-medial direction, both in terms of the magnitude of forces exerted and in terms of time synchrony, could be responsible for knee and ankle injuries due to the possible occurrence of compensatory mechanisms carried out by the participants during pedalling in order to maintain the conditions of workload and cadence.^18^

## Conclusions

In this work, an analysis of asymmetries in the three-dimensional forces applied to the pedals during pedalling has been carried out. The forces applied to the pedals were recorded using devices previously developed by the authors.

The NSI index has been used to analyse the asymmetry level between the different pedalling force components in both legs. The interpretation of the results provided by the NSI caused confusion when a negative NSI was obtained and the force in the dominant leg was greater in modulus than the force applied by the non-dominant leg. In this case, the need to complement the NSI study with the values of the forces performed at each instant for a correct and global interpretation of the results has been shown. An index (ADC) has been defined from the NSI to quantify the percentage of the pedal cycle in which a force component is asymmetric. Analysis of results suggests that the components of the forces applied to the pedal contained in the sagittal plane are predominantly symmetrical throughout the pedal cycle. However, the lateral-medial component of the force is asymmetric in a high percentage of the pedal cycle.

The use of this index was complemented by the CCC calculation to analyse similarities in the applied force patterns and possible time lags between the patterns in the dominant and non-dominant legs. The joint use of the NSI and CCC coefficients allows establishing a methodology to more accurately and completely analyse existing asymmetries in pedalling forces. The obtained results suggest that these asymmetries are not only due to differences in the forces applied by each leg, but also that the pattern of forces exerted by each leg during the cycle also influences. These preliminary results must be corroborated by carrying out a study with a larger sample*.* The results obtained reflected that the tangential and radial forces showed a high level of symmetry during the pedalling cycle, which was consistent with previously published analyses of symmetry at the kinematic level. However, as future work, an analysis of muscle forces during pedalling would be interesting to study possible asymmetries at a muscle level. Previous studies suggested that symmetries at the kinematic and dynamic level can be achieved through compensation mechanisms involving a high level of asymmetry in muscle forces, which would imply a high risk of pain and injury.

The results obtained in the lateral-medial direction showed the most asymmetrical behaviour. This fact was related to the low levels of force performed in this direction and the difficulty, at a biomechanical level, of controlling it. In addition, the participants were all amateurs. A study with professional cyclists will be of interest in order to analyse the level of dispersion and asymmetry obtained in this force component. However, the obtained results were consistent with previous kinematic studies which concluded that results in planes different from the sagittal plane showed a higher asymmetry level.

The asymmetry level presented in the lateral-medial force component was also shown as a lower correlation between the force patterns in both legs, as well as a lag between the two patterns. Preliminary studies suggested that this advance may be related to low workload and cadence where inertial forces would play an important role. A relationship between workload and the pattern lag between dominant and non-dominant leg may exist. Future studies were planned to develop this line of research further.

The results obtained in this work highlighted the importance of three-dimensional analysis in cycling dynamics. The forces applied outside the sagittal plane were found to be an order of magnitude lower than those applied in the sagittal plane. However, the asymmetry level presented in the lateral-medial direction motivated the three-dimensional dynamic analysis in pedalling in order to identify possible causes of pain or injury produced by this asymmetric behaviour.
